# All-in-one aerial image enhancement network for forest scenes

**DOI:** 10.3389/fpls.2023.1154176

**Published:** 2023-03-28

**Authors:** Zhaoqi Chen, Chuansheng Wang, Fuquan Zhang, Ling Zhang, Antoni Grau, Edmundo Guerra

**Affiliations:** ^1^ College of Computer and Big Data, Fuzhou University, Fuzhou, China; ^2^ Fujian Provincial Key Laboratory of Information Processing and Intelligent Control, Minjiang University, Fuzhou, China; ^3^ Department of Automatic Control, Polytechnic University of Catalonia, Barcelona, Spain; ^4^ College of Computer and Control Engineering, Minjiang University, Fuzhou, China; ^5^ Digital Media Art, Key Laboratory of Sichuan Province, Sichuan Conservatory of Music, Chengdu, China; ^6^ Fuzhou Technology Innovation Center of Intelligent Manufacturing information System, Minjiang University, Fuzhou, China; ^7^ Engineering Research Center for Intangible Cultural Heritage (ICH) Digitalization and Multi-source Information Fusion (Fujian Polytechnic Normal University), Fujian Province University, Fuzhou, China

**Keywords:** image enhancement, all-in-one network, multi-receptive fields, drone image monitoring, forest protection, smoke detection

## Abstract

Drone monitoring plays an irreplaceable and significant role in forest firefighting due to its characteristics of wide-range observation and real-time messaging. However, aerial images are often susceptible to different degradation problems before performing high-level visual tasks including but not limited to smoke detection, fire classification, and regional localization. Recently, the majority of image enhancement methods are centered around particular types of degradation, necessitating the memory unit to accommodate different models for distinct scenarios in practical applications. Furthermore, such a paradigm requires wasted computational and storage resources to determine the type of degradation, making it difficult to meet the real-time and lightweight requirements of real-world scenarios. In this paper, we propose an All-in-one Image Enhancement Network (AIENet) that can restore various degraded images in one network. Specifically, we design a new multi-scale receptive field image enhancement block, which can better reconstruct high-resolution details of target regions of different sizes. In particular, this plug-and-play module enables it to be embedded in any learning-based model. And it has better flexibility and generalization in practical applications. This paper takes three challenging image enhancement tasks encountered in drone monitoring as examples, whereby we conduct task-specific and all-in-one image enhancement experiments on a synthetic forest dataset. The results show that the proposed AIENet outperforms the state-of-the-art image enhancement algorithms quantitatively and qualitatively. Furthermore, extra experiments on high-level vision detection also show the promising performance of our method compared with some recent baselines.

## 1 Introduction

Drone aerial image technology plays an indispensable role in forest fire monitoring. However, the images captured by drones are severely damaged because of the uncertainty and instability of aerial photography. Typical examples of aerial image degradation include atmospheric interference and motion blur caused by the vibration of the drone. Moreover, the aerial images could further suffer from the visual impact of compression when the images are transmitted back through the network. Therefore, how to restore degraded aerial images is particularly significant under the limitation of existing hardware. Recently, with the development of deep learning, data-driven methods designed for task-specific image enhancement have achieved great success, such as image dehazing (Ren et al., 2018; Qu et al., 2019; Wang et al., 2020; Song et al., 2022), image denoising (Zhang et al., 2017b; Ct et al., 2020), and image deblurring (Nah et al., 2017; Gao et al., 2019). However, an all-in-one image enhancement model seems more effective than its specific-task counterpart in practical application scenarios as real-world images usually suffer various degradations. For example, images of forest scenes collected by drones could be affected by adverse weather or blurred by remote sensor shaking. In contrast, integrating multiple image enhancement tasks in an all-in-one framework is a promising choice.

Recently, Li et al. proposed an all-in-one method, which uses a multi-encoder and single-decoder architecture to address various weather corruptions (Li et al., 2017). It also utilizes the neural architecture search to optimize the features extracted by the encoder, which performs better than previous task-specific image enhancement algorithms. But, designing such an architecture usually comes at the expense of computational costs. Due to its success in high-level tasks such as image classification, segmentation, and detection, the transformer has been used in low-level vision tasks. Valanarasu et al. proposed Transweather, an end-to-end multi-weather image restoration model, as an alternative solution to multi-encoders for the same application scenario (Valanarasu et al., 2022). Li et al. also proposed a unified framework capable of recovering images with unknown degradation types, which has demonstrated its effectiveness in image enhancement affected by natural weather (Li et al., 2022). Although the generalization performance of the network has been verified on multiple datasets, it has low practical application value due to its large number of parameters and computational delays. Moreover, nearly all of the representative models for aerial image enhancement are based on single-task design (Wang and Liu, 2022). Therefore, research on an all-in-one framework is still very necessary in this field.

**Figure 1:**
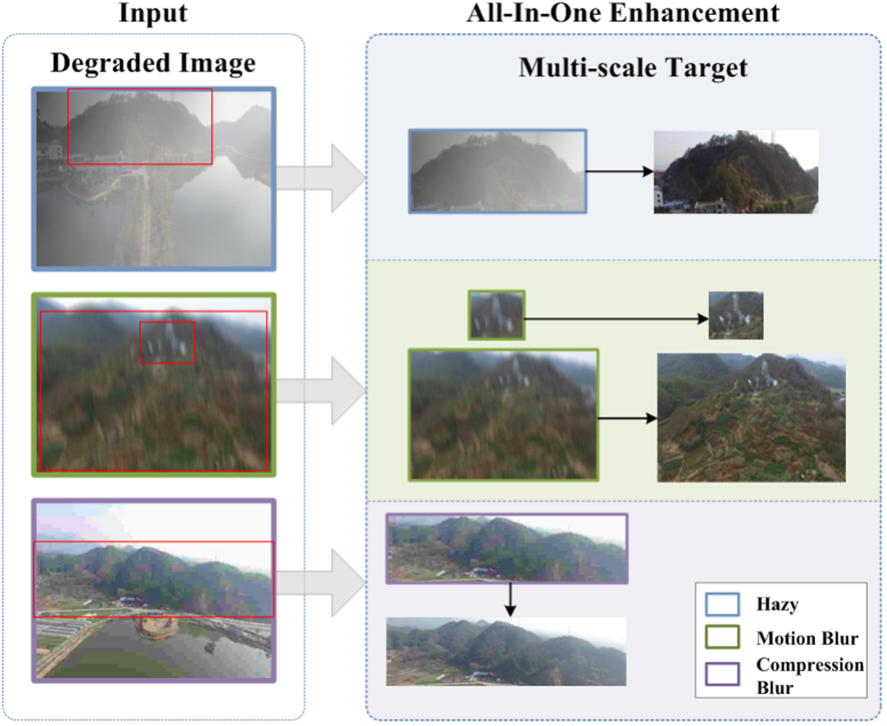
An illustration of our motivation. As shown, the forest scene images captured by drones could inevitably suffer from different degradation problems. The target size in the images captured at different locations is variable. Therefore, we explored the restoration of multi-scale target details in aerial images and proposed an all-in-one image enhancement method.

We believe that the future development of aerial image enhancement research lies in all-in-one models, which is also a critical step toward general technology research. The motivation for this paper is two-fold: on the one hand, we wish to conduct an in-depth study on preserving the high-dimensional detail features of multi-scale objects, thus pushing the aerial image reconstruction methods to a new level. On the other hand, the all-in-one network can be utilized to study general strategies for a seamless transition between different tasks and domains. As shown in **Figure 1**, to this end, we propose an All-in-one Image Enhancement Network (AIENet) based on a Multi-Receptive Field (MRF) enhancement block. Specifically, the model only performs one downsampling operation on the original image. And the global skip connection is used to introduce the low-level feature information of the corresponding scale into the deconvolution process so that the model can obtain more high-resolution details during upsampling. In addition, with the multi-receptive field enhancement module, the model can fully use the prior hierarchical features on the same scale to explore different regions and then obtain the global context by aggregating the context information collected from different areas.

The main contributions of this work include the following:

- By comprehensively analyzing the characteristics of aerial imagery, we identify the importance of all-in-one models for forest scenarios. Furthermore, we accurately reconstruct local textures and microstructures in degraded images by maximizing the feature representation and learning capabilities of neural networks, thereby improving the accuracy of subsequent high-level computer vision tasks.
- We propose a lightweight image enhancement model AIENet, which can quickly solve the degradation problem in an all-in-one framework when collecting images. The proposed method utilizes the global and local skip connections to introduce high-resolution details into the output image. And the model designed in this paper cleverly uses the multi-receptive field fusion technique to perceive the same feature map from multiple scales, thus making up for the insufficient ability to capture global image features.
- We demonstrate that our method can achieve better visual performance and high scores based on the quantitative metrics in task-specific and all-in-one aerial image restoration on the forest wildfire dataset. The model proposed in this paper also provides an idea of integrated processing for restoring the visual quality of images with complex scenes.

The remainder of this paper is organized as follows. Section 2 discusses related work on image enhancement and multi-receptive field technology. The proposed method is introduced in Section 3. Section 4 analyzes the comparative experimental results, and our work is concluded in Section 5.

## 2 Related works

With the popularity of graphics processing units, the deep learning approaches (Alsubai et al., 2022; Farghaly et al., 2022) have developed the most advanced model in the computer vision field, and numerous elegant solutions (Xue et al., 2019; Xue et al., 2021) have been proposed for visual tasks in the last few years. In the field of image enhancement, most researchers work on task-specific image restoration. In this paper, we innovatively propose an all-in-one architecture to solve the image degradation problems encountered in various stages of aerial image acquisition, such as haze weather interference, the vibration of the remote sensing platform during shooting, and image compression during transmission distortion. Therefore, we first describe representative methods for each task. Then, we introduce related work on multi-scale receptive fields in low-level vision.

### 2.1 Image enhancement

**Image Dehazing:** Since Mccartney et al. proposed the atmospheric scattering model to approximate the haze effect which is shown as: $\hat{x}=x⊙t+A⊙(1-t)$ , where $\hat{x}$ and *x* mean the degradative images and restored images, respectively. *t* is the transmission map, which can express as: *t* = *e* ^-β^ *^d^*, where β and *d* are the scattering coefficient and depth map (McCartney, 1976). ***A*** is the global atmospheric light and ⊙ represented as pixel-wise multiplication. Recent image dehazing methods could be classified into two families, *i.e.*, prior-based methods and learning-based methods. In traditional prior-based methods, many image statistical priors are used as additional constraints to compensate for information loss during image degradation. He et al. proposed a classic image dehazing method that depends on the statistical results called Dark Channel Prior (DCP), which generates at least one low-intensity pixel in the color channel of each pixel local neighborhood (He et al., 2010). Then the learned transmission map is used to calculate the haze-free image through the physical model. Wang et al. found that the blurred areas are mainly concentrated on the brightness channel of the YCrCb color space (Wang et al., 2018) Therefore, it is possible to enhance the visual contrast of foggy scenes by recovering the missing texture information in the luminance channel. As for learning-based methods, the techniques such as attention (Liu et al., 2019; Zhang et al., 2020), feature fusion (Dong et al., 2020; Qin et al., 2020) and contrastive learning (Wu et al., 2021; Chen et al., 2022) are widely used to improve single-image dehazing performance. Moreover, they outperform the traditional prior-based image dehazing methods.

**Motion Deblurring:** Since large-scale real-world blur data is challenging to obtain, most traditional deblur methods are generally tested on synthetic images from $\hat{x}$ to *x*, which can be expressed as $\hat{x}=x⊗k+n$ , where $\hat{x}$ is the blurred image generated from clean image *x*, *k* is the blur kernel or convolution kernel, ⊗ denotes the convolution operator and *n* is additive noise. However, handcrafted methods are not good at capturing complex blur variations in authentic images. In contrast, CNN-based methods can handle real-world blurry images well if we have a dataset of paired images. Tao et al. proposed a multi-scale approach based on encoder-decoder recurrent networks (SRN), which is the first method to integrate recurrent neural networks (RNN) into deblurring models (Tao et al., 2018). Some methods (Kupyn et al., 2018; Kupyn et al., 2019) based on Generative Adversarial Networks (GAN) have also achieved competitive results on real-world deblur. Recently, multi-stage architecture networks (Chen et al., 2021; Zamir et al., 2021) have achieved state-of-the-art results in deblurring restoration tasks.

**Compression Deblurring:** Early image compression restoration methods use deblocking filters to reduce discontinuities between pixel blocks. To reduce blocking artifacts in compressed images, Lee et al. adaptively use various block predictors based on frequency components in the Discrete Cosine Transform (DCT) domain (Lee et al., 2004). Yoo et al. classifies blocks as flat or edge blocks and applies different deblocking filters depending on the classification result (Yoo et al., 2014). However, these methods employing deblocking filters only target blocking artifacts. But also other artifacts in compressed images, such as ringing artifacts. Therefore, most scholars have conducted extensive research on CNN-based compression deblur. Dong et al. introduce a super-resolution convolutional neural network for reducing compressed image artifacts (Dong et al., 2015). Zhang et al. use auto-encoders in both DCT and pixel domains, considering the output of auto-encoders and input images to reduce visual artifacts in compressed images (Zhang et al., 2018). Lee et al. utilize parallel atrous convolution residual blocks to extract a variety of features with large receptive fields, then use attention mechanism for the output of atrous convolution to obtain representations of the global region (Lee et al., 2021).

**All-in-One Image Enhancement:** Although the above image enhancement methods all perform well on specific tasks, real-world images are often easily corrupted by different degradation types, making task-specific image enhancement lack flexibility and generalization in practical applications. Recently, some work has focused on all-in-one visual enhancement networks. To deal with image degradation under severe weather conditions (such as rain, haze, and snow), Li. et al. present an ensemble model based on neural architecture search, whose generator has a multi-encoder and a typical decoder architecture (Li et al., 2020). In other words, the network must train different models for different degradation problems, which is unsuitable for an all-in-one solution in practical applications. Most recently, Valanarasu. et al. propose an alternative state-of-the-art solution to this problem with TransWeather (Valanarasu et al., 2022). As an end-to-end vision transformer (Dosovitskiy et al., 2021) based multi-weather image restoration model, it exhibits more powerful versatility. Notably, these two all-in-one image enhancement methods focus on recovering the same combination of degradation types (i.e., weather disturbances). However, solving the image degradation problem under multiple conditions (such as weather, physical factors, etc.) in an all-in-one framework can better meet the most practical scenes. In addition, the real-time requirement of the remote sensing platform also means that the model design should be simplified. In this paper, we only use simple tricks to capture the global degradation representation of blurred images, building an all-in-one framework for handling different degradation types.

In summary, most image enhancement methods are designed for specific types of degradation, making it difficult to generalize to other image enhancement tasks. For example, state-of-the-art image dehazing methods typically rely on the atmospheric scattering physical model to recover images by estimating unknown parameters in the physical model. Similarly, image motion deblurring methods are usually designed based on linear motion blur physical models. Research on image compression deblur typically focuses on removing artifact blocks. Compared to these task-specific image enhancement methods, our model can restore images of different degraded types, which effectively alleviates the shortage of storage resources in complex application scenarios. Moreover, existing all-in-one image enhancement approaches focus on the study of image degradation caused by severe weather (*e.g.* haze, rain or snow), while our work committed on image degradation caused by different factors (*e.g.* haze, motion blur or compression blur). Notably, they usually insert modules such as transformers or attention mechanisms into the network, which can easily introduce a large number of parameters that make it difficult to meet practical requirements. Therefore, we devise an all-in-one model with characteristics of lightweight and obtain blur-free aerial images characterized by good visibility that is more responsive to practical scenarios.

### 2.2 Receptive field in low-level vision

The receptive field in the deep neural network represents the size of the area mapped on the original image by the pixels on the output feature map of each convolutional layer. Since the network generally uses convolutional and pooling layers which are locally connected, neurons cannot perceive all the characteristics of the original image. Therefore, Zhang et al. employ dilated filters to expand the receptive field (Zhang et al., 2017a). However, dilated filter inherently suffers from grid effects, where the receptive field only considers a sparse sampling of the input image with a checkerboard pattern. To avoid the increased computational burden and potential sacrifice of performance improvement, Liu et al. expand the receptive field by applying a wavelet transform to the U-Net architecture and propose a multi-layer wavelet CNN (MWCNN) model with reduced computational complexity (Liu et al., 2018). Fu et al. propose deep convolutional sparse coding architecture with atrous convolution to obtain a high-level receptive field (Fu et al., 2019). Although these methods are able to ensure that the neurons cover the image area entirely. However, an excessively large receptive field easily introduces redundant information to the small target area, which reduces the performance of the model. To solve the problem of differences in the distribution of target regions in aerial images, this paper uses parallel convolution with different convolution kernels to extract multi-scale target region features, so as to obtain a more effective global degradation representation.

## 3 Method

**Figure 2:**
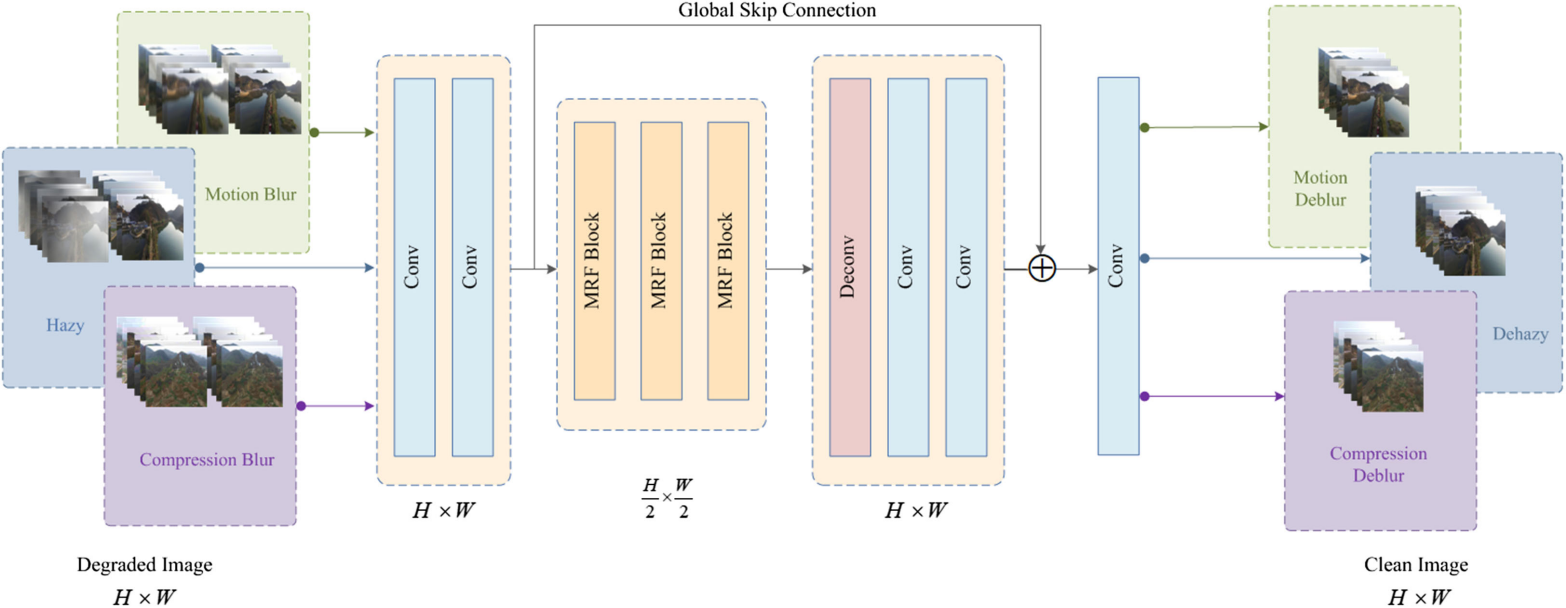
The overall structure of our All-in-one Image Enhancement Network (AIENet) which addresses image hazy, motion blur, and compression blur problems in an all-in-one model.

In this section, we elaborate on the architecture of the proposed all-in-one image enhancement network AIENet. The overall architecture of the model is shown in **Figure 2**. The model can strike a balance between speed and accuracy. Given a degraded image, AIENet first performs a unique downsampling operation. Subsequently, to yield a more effective and comprehensive degraded representation, we adopt multiple receptive fields, catering to a wide range of target region sizes. Lastly, the global skip connection is used to fill in the blank content of the deconvolution process to get purer high-resolution information. To showcase the competence of the proposed model, we present three typical image degradation problems encountered by drones when monitoring forest landscapes, namely haze, motion blur, and compression blur, as targeted examples in this paper. In the following sections, we first illustrate the multi-receptive field image enhancement block, which forms the fundamental component of AIENet, and then elaborate on the overall model architecture featuring a skip structure. Finally, the objective function of the model is discussed.

### 3.1 MRF enhancement block

**Figure 3:**
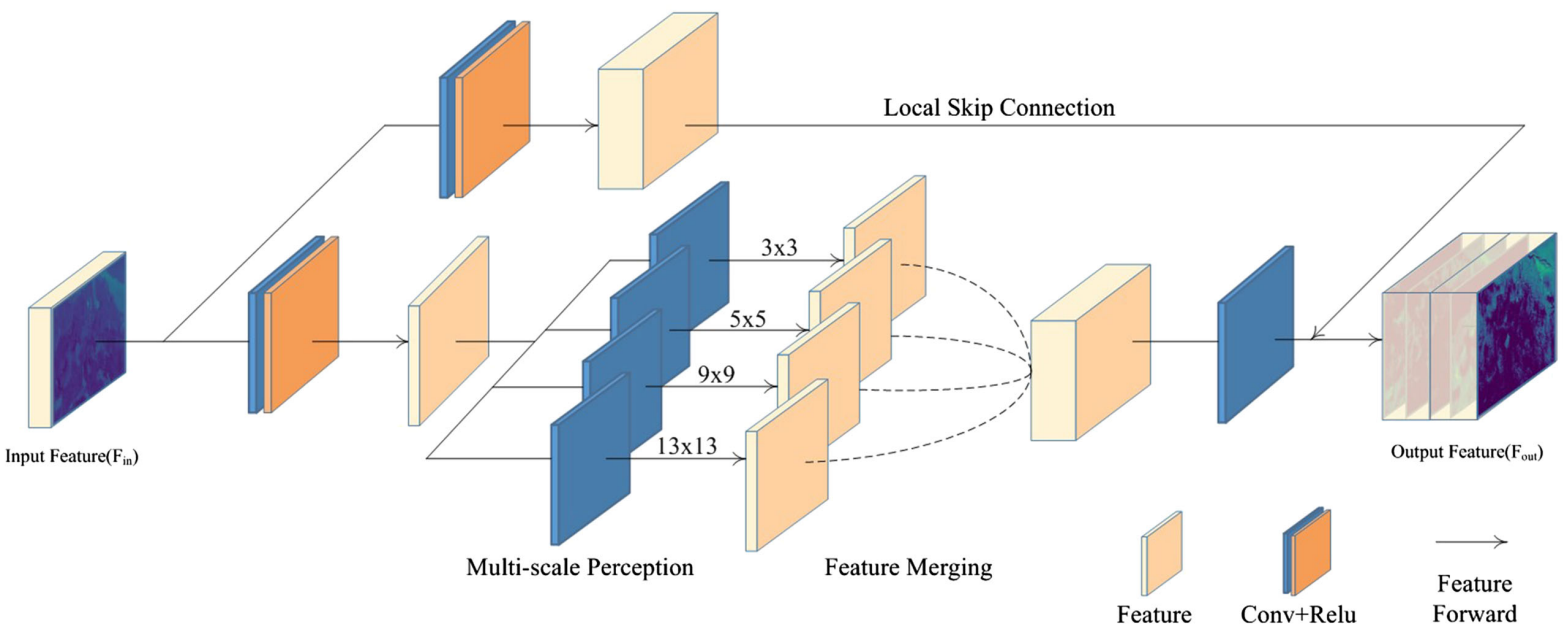
The MRF enhancement block comprises two branches: the high-dimensional extract branch and the multi-scale perception branch. The high-dimensional extract branch is responsible for preserving the high-resolution details of the input features, while the multi-scale perception branch employs convolution kernels of varying sizes to capture multi-scale features.

The MRF enhancement block is a versatile module with a plug-and-play design, enabling its integration into any part of an existing network. Notably, this block offers multi-scale area perception, guaranteeing the inclusion of various scale feature details in the final outcome. It can be decomposed into two fundamental components: 1) a multi-scale perception module, responsible for extracting distinct scale representations; 2) a feature merging operator, which merges intermediate feature maps. Specifically, the features of the last layer are initially fed into two distinct branches, each engaging in feature extraction *via* diverse dimensions. The multi-scale perception refers to the lower-dimensional branch within the block, which employs convolution kernels of varying sizes to facilitate multi-scale feature perception. The enhancement block concludes by utilizing channel-wise concatenation, which enables the learning of comprehensive contextual information. We elaborate on these processes in detail below. The pipeline of the MRF enhancement block is shown in **Figure 3**.

#### 3.1.1 Multi-scale perception

The receptive field in a convolutional neural network represents the visual range of the network with respect to the input image. As only the input pixels within the receptive field contribute to the calculation, the size of the receptive field can be used to measure the ability of the model to leverage spatial information. However, it is not always optimal for the receptive field size to be maximized. In the case of larger targets, a larger receptive field can better integrate contextual information about the target area and restore its high-resolution details. For smaller objects, a larger receptive field can easily introduce excessive artifacts that may hinder the image restoration process. Especially for aerial images that are captured from multiple angles, the scale of the target area can constantly expand or shrink depending on the position of the drones. It is worth noting that a single receptive field may not always yield optimal results in learning the complex scale structures of aerial images. As such, Ren et al. and Liu et al. proposed a solution for multi-scale feature extraction Ren et al. (2016); Liu et al. (2019). While the design of multi-scale stacking allows the network to have a larger expression space in the receptive field, the network’s receptive field is fixed in the inference stage when the model parameters are not updated. This is the statistical receptive field calculated by the model based on the data distribution of the training set, which may be suboptimal for each specific image. Additionally, by extracting intermediate features through this concatenated approach, gradient vanishing may occur, and the signal generated in earlier iterations may be disrupted.

To effectively address the aforementioned issues, this paper proposes the generation of intermediate feature maps through distinct branches. The aim of multi-scale perception is to utilize diverse receptive fields to enhance the comprehension of various regions and acquire multiple global degraded representations. Indeed, it is possible to design the multi-scale perception module to be highly complex, maximizing the reasoning ability of the model. However, even with a simple parallel usage of several convolutional layers with different kernels and the use of skip connections to concatenate the shallow features with these multi-scale perception feature maps, we can already intuitively observe the efficiency of feature extraction under multi-scale perception. Specifically, in order to better preserve the high-resolution information of the original image, the resolution of all intermediate feature maps in the enhancement block is kept consistent with that of the input feature map. Formally, let’s define *F_in_* ∈*R^H×W×C_in_^* as the input feature map generated by the last layer. The enhancement block initially feeds the input feature map *F_in_* into a dual-branch structure for feature extraction. The high-dimensional extract branch aims to learn more comprehensive original image features by expanding the channel dimension. The operation on the input feature map *F_in_* can be defined as follows:


(1) $$F_{s}=\text{Relu }\left({\text{Conv}}_{c_{1}}(F_{in})\right)$$

Where $Conv_{c_{1}}\cdot (\cdot )$ is the convolution layer using *c*_1_ convolution kernels and *F_s_* ∈*R^H×W×C^*_1_. Nevertheless, the local information captured by each pixel in *F_s_* is limited. To address this issue, the multi-scale perception branch adopts a multi-kernel strategy, consisting of four convolutional layers with different kernels to capture more diverse features. This trick enables the network to conduct intricate feature extraction operations in low-dimensional feature spaces and reduce the number of model parameters. The resulting feature map generated by the parallel convolutional layers can be represented as follows:


(2) $$F_{d}=\text{Relu }\left({\text{Conv}}_{c_{2}}(F_{in})\right)$$

(3) $$f_{i}=\omega_{i}{\left(F_{d}\right)}_{H\times W\times C_{2},}\;i\;\in \;\{1,2,3,4\}$$

where ω*_i_* (·) denotes the parallel convolutional operation that generates the *i^th^* scale receptive field and *F_d_* ∈*R^H×W×C_2_^*. This dual-branch structure maintains a relatively independent calculation scheme. The combination of a “deep network + multi-scale feature extraction” and a “shallow network + high-dimensional feature extraction” takes into account both the rich details of shallow features and the abstract semantics of multi-scale features. This combination also effectively manages the computational overhead to ensure the real-time performance.

#### 3.1.2 Feature merging

Once the input feature map passes through multiple receptive fields of feature perception, as much contextual information as possible has been constructed between these different scales of receptive fields. We observe that for spatial tasks such as image enhancement, parallel multi-scale capabilities are required to handle perceptually large and small objects. In traditional processing methods, there are primarily two approaches for merging different feature maps: concatenation and element-wise addition. The latter requires the feature maps to have identical channels, necessitating the conversion of feature maps to uniform channels. However, this requirement restricts the flexibility of merging feature maps and direct summation of all feature maps may remove the generated image details. Instead, we adopt the concatenation approach to aggregate features from different receptive fields. At this stage, the feature channels already contain various local contextual information adapted to the target area size. The merged features can be further abstracted through convolution, allowing for soft transitions between receptive fields of varying scales and facilitating the construction of a holistic global context. To better convey the semantic details of the input features during training, skip connections are employed to ensure the effectiveness of the enhancement block, resulting in an output feature map with high-dimensional details and multi-scale perception. Let’s define *F_i_* as the feature map generated by the *i^th^* MRF enhancement block. The output feature map can be expressed as:


(4) $$F_{m}=\text{ Cat }\left({\text{Conv}}_{c_{1}}(Cat(f_{i})),F_{s}\right),\;i\;\in \;\{1,2,3,4\}$$

where *Cat* (·) indicates channel-wise concatenation operator. In this way, feature maps of arbitrary numbers can be merged, which gives the MRF the potential to capture more details of multi-scale target areas.

### 3.2 Global-local skip connection

The residual network architecture has exhibited outstanding performance in computer vision tasks spanning from low-level to high-level tasks(Kim et al., 2016; Dong et al., 2020). This architecture was initially proposed by He et al. for image recognition (He et al., 2016). The purpose of the skip connection is to merge low-level features and high-level convolutional features with more intricate semantics. In spatial feature reconstruction tasks like image enhancement, the rich details preserved by high-level convolutions are extremely valuable. Nonetheless, the increase in the receptive field with network depth may result in the loss of high-dimensional details. To maintain fine details from the input image to the output image, we incorporate local skip connections, which significantly enhance the performance. To be more precise, the features extracted from the previous layer are first processed through convolution for high-dimensional feature extraction, and then combined with the multi-receptive field features before being passed on to the next module. This approach reduces the susceptibility of the model to loss of high-frequency information that may occur due to repeated convolution operations.

Although Liu et al. and Gao et al. successfully applied skip connections to image enhancement problems (Liu and Yang, 2018; Gao et al., 2019), it should be noted that deconvolution or upsampling often requires filling in a significant amount of missing content. It is important to acknowledge that generating high-quality results from scratch requires sufficient auxiliary information. To address the issue, we devise a novel skip connection that can take into account both global and local contextual information interaction. By employing a cross-layer global skip connection, the corresponding scale features are introduced into the deconvolution or upsampling process, which can effectively preserve high-resolution details contained in the input images. This results in an enhanced ability of the network to recover image details, as illustrated in **Figure 2**. To exploit the merits of both designs, the model contains *n* MRF enhancement blocks and a global skip connection. Each enhancement block comprises local skip connections that fuse high-dimensional detail and multi-receptive field features. Such a residual structure allows the network to train deep models without sacrificing shallow information features.

### 3.3 Model architecture and loss function

Following the similar network design principle in Cai et al., we also design the overall network as a simple auto-encoder, where three residual blocks are inserted between the encoder and decoder to enhance its understanding capacity of different target regions (Cai et al., 2016; Ren et al., 2016; Li et al., 2017). Specifically, two convolutional layers are first used to encode the input blurred image into the feature map. This feature map is used as the encoder part, where only the last convolutional layer downsamples the feature map by a factor of 1/2. Correspondingly, a deconvolutional layer with a stride of 1/2 is used in the decoder part to upsample the feature maps to the original resolution. The feature maps are subsequently transformed back to image space using three convolutional layers to obtain the final blurred residual. For the middle residual block, we call it a “multi-receptive field enhancement block”, because it uses four convolution kernels of different sizes to extract the details of varying target areas adaptively. The sizes of the four convolution kernels are set as 3x3, 5x5, 9x9, and 13x13, respectively. To obtain a good trade-off between performance and running time, we set the number of channels of all intermediate convolutional layers in the enhancement block to 32 or 128. Then an instance normalization (Ulyanov et al., 2016) and a Relu layer are placed after each convolutional layer. Each layer setting for the network is given in **Table 1**. Fan et al. has proved that in addition to the input image, pre-calculating the edges of the input image and feeding it into the network as auxiliary information is beneficial for network learning. By default, we also adopt this idea and concatenate the pre-calculated edges with the input blurred image along the channel dimension as the final input of the network (Fan et al., 2017; Fan et al., 2018; Ren et al., 2018).

**Table 1:** Network Setting.

	Layer Description	Output Size
Encoder		
#1	Conv (3, 32, 3, 1)	640x640x32
#2	Conv (32, 64, 3, 2)	320x320x64
3x MRF Enhancement Block(c)		
#1_1	Conv (c, 32, 3, 1)	320x320x32
#1_2	Conv (c, 128, 3, 1)	320x320x128
#2_1	Conv (32, 32, 3, 1)	320x320x32
#2_2	Conv (32, 32, 5, 1)	320x320x32
#2_3	Conv (32, 32, 9, 1)	320x320x32
#2_4	Conv (32, 32, 13, 1)	320x320x32
#3	Cat + Conv (128, 128, 3, 1)	320x320x128
Decoder		
#1	Deconv (256, 128, 4, 2)	640x640x128
#2	Conv (128, 64, 3, 1)	640x640x64
#3	Conv (64, 32, 3, 1)	640x640x32
#4	Conv (64, 32, 3, 1)	640x640x3

Where c indicates the number of feature channels entering the MRF enhancement blocks.

Most learning-based image enhancement methods (Cai et al., 2016; Ren et al., 2016; Li et al., 2022) use Mean Square Error (MSE) loss to train the models. Following the same strategy, we also use this simple loss. Specifically, we adopt the strategy of (Ren et al., 2018) and set the learning objective of the model as the residual between the clear image and the input degraded one. In summary, the total loss can then be written as follows:


(5) $$L={\left\|\hat{r}-r\right\|}^{2}$$

where $\hat{r}$ is the predicted residual, *r* is the residual of the degraded image and clear image at location (*i*,*j*), which can be calculated as follows:


(6) $$r=\sum_{i=1}^{H}\sum_{j=1}^{W}\left(h(i,j)-g(i,j)\right)$$

Even with the only simple loss mentioned above, our method can still achieve state-of-the-art performance on aerial image enhancement. Further, this kind of loss function also enables efficient training due to the smaller number of parameters to update.

## 4 Experiments

This section provides qualitative and quantitative comparisons with state-of-the-art methods for three challenging aerial image enhancement tasks, *i.e.*, image dehaze, image motion deblur, and image compression deblur. We first introduce the dataset source and experimental settings. Then, we present the results of comparing our proposed algorithm with 15 state-of-the-art methods. Finally, the effectiveness of the proposed module is demonstrated through ablation experiments.

### 4.1 Dataset

We constructed datasets of degraded aerial images to evaluate the effectiveness of the proposed method in handling various types of image degradation. The raw images were sourced from a publicly available dataset, which comprised video frames captured by drones equipped with video surveillance cameras. To obtain degraded images of drone-monitored forest scenes under various conditions, we employ Python library Imgaug (Jung et al., 2020) to synthesize paired degraded images. For the 2007 original images, we generated two degraded images with different levels of degradation for each image by adjusting different parameters depending on the degradation type. In this paper, we synthesize haze images with different concentrations by setting the scattering coefficient to 2 or 3, generate motion blur images using blur kernels ranging from 25 to 34 and angles ranging from -150 to 360 degrees, and produce compression blur images by randomly selecting compression ratios between 89 and 93. Regarding the scattering coefficient used in generating hazy images, we based them on previous research about the atmospheric scattering physical model. The motion blur kernel and angle parameters were selected based on the linear motion blur physical model. The compression rate parameters are based on the pixel count of the image and the compression algorithm parameters. Finally, 4014 degraded images were generated for each of the three tasks, 70% of which are used for fully supervised training and the rest for testing. Since we use a mixture of three degraded types in the all-in-one image enhancement task, our all-in-one framework can effectively generate close-to-ground truth images for any degradation type.

### 4.2 Training specifications

**Figure 4:**
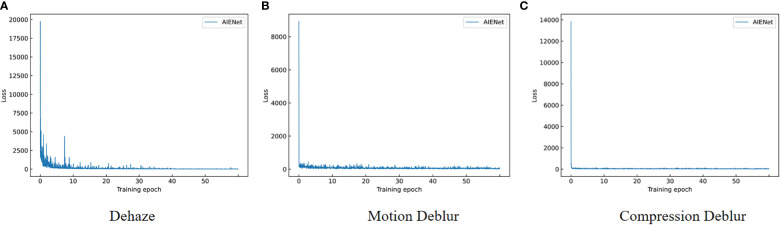
Curves of the changing trend of target loss. **(A)** shows the trend of the dehazing loss function, **(B)** shows the trend of the compression deblurring loss function, and **(C)** shows the trend of the motion deblurring loss function.

All experiments are conducted using PyTorch on an Ubuntu 20.04 system, with NVIDIA RTX 3080Ti GPU to optimize the training speed. For each task, we compare the proposed method with the state-of-the-art methods separately. Then the generality of AIENet is demonstrated by further comprehensive training. We use almost the same training strategy for these models. For a fair comparison, all models are trained for 60 epochs. By default, we train our model with batch size 2 using the Adam optimizer (Kingma and Ba, 2014). The default initial learning rate is set to 0.001, decaying by 0.1 every 40 epochs. The changing trend of target loss is shown in **Figure 4**. In the early stages of training, the loss value is relatively high, indicating a large discrepancy between the predicted and ground truth images. However, the loss value of the model drops very quickly after training several epochs and plateaus at epoch 40. This suggests that our model may have converged earlier, but we still follow the default training strategy for comparison with previous work.

### 4.3 Comparisons with state-of-the-art methods

Our model is individually compared with five state-of-the-art methods on specific tasks for a comprehensive comparison. Specifically, we compare with (Li et al., 2017; Ren et al., 2018; Dong et al., 2020; Qin et al., 2020; Li et al., 2021) on the dehazing task. The methods to remove motion blur include (Nah et al., 2017; Tao et al., 2018; Kupyn et al., 2019; Zhang et al., 2019; Cho et al., 2021). The baselines for compression deblur are (Dong et al., 2015; Chang et al., 2020; Chen et al., 2021; Jiang et al., 2021; Zamir et al., 2021). To demonstrate the superiority of our all-in-one framework, we also compare models trained in an all-in-one manner on three tasks. In other words, we train the proposed model on an ensemble of all datasets consisting of degraded images with three different degradation types (i.e., haze, motion blur, and compression blur). And then test on a single type.

Quantitative evaluations between ground truth *x* and restored images *y* were performed *via* the conventional Peak Signal-to-Noise Ratio (PSNR) (Huynh-Thu and Ghanbari, 2008) and Structural Similarity (SSIM) (Wang et al., 2004) metrics. PSNR is a very important indicator in the field of image enhancement, which can be expressed as:


(7) $$PSNR=10\log_{10}\left(\frac{L^{2}}{MSE}\right)$$

where *L* is the possible maximal pixel value. The mean square error *MSE* between *x* and *y* is calculated as follows where *H* and *W* are the height and width of the images:


(8) $$MSE(x,y)=\frac{1}{H\times W}\sum_{i=1}^{H}\sum_{j=1}^{W}{\left(x(i,j)-y(i,j)\right)}^{2}$$

In comparison to PSNR, the structural similarity indicator is more in line with human subjective system judgment on image quality. SSIM is designed to compute the luminance, contrast, and structural similarity between the *x* and *y*, which can be represented by:


(9) $$SSIM(x,y)=\frac{\left(2{\text{μ}}_{x}{\text{μ}}_{y}+c_{1})(2{\text{σ}}_{xy}+c_{2}\right)}{\left({\text{μ}}_{x}^{2}{\text{+μ}}_{y}^{2}+c_{1})({\text{σ}}_{x}^{2}{\text{+σ}}_{y}^{2}+c_{2}\right)}$$

where μ*_x_* and μ*_y_* are the mean of *x* and *y*, respectively. σ*_x_* and σ*_y_* are the variance of *x* and *y*, respectively. σ*_xy_* is the covariance of *x* and *y*. By default, *c*_1_=·(0.01*L*) and *c*_2_=·(0.03*L*) are the constants used to avoid divisors by zero. We evaluated PSNR and SSIM based on the luminance channel Y of the YCbCr color space in accordance with the previous convention (Zamir et al., 2021; Valanarasu et al., 2022).

#### 4.3.1 Task-specific image enhancement results

**Quantitative Evaluation for Image Enhancement.** **Table 2** presents our quantitative evaluations. The top half of the tables contain results from task-specific image restoration. Our models achieve performances superior to all compared existing methods in PSNR on all tasks. For the image dehazing task, the proposed method yields the best PSNR of 35.69 dB, which also outperforms all dedicated to dehazing models. Notably, in our experiments, we found GCANet (Ren et al., 2018) to be the best-performing network for dehazing in SOTAs. And the method in this paper also achieves a breakthrough of 5.37%. Furthermore, we also get small victories in objectively evaluating SSIM close to the Human Visual System (HVS). In the motion deblurring task, our model exceeds all compared deblurring networks in terms of PSNR. It is worth noting that our model is the second best in the comparison of structural feature recovery. But compared to MIMO-UNet (Cho et al., 2021), the best network for motion blur removal in this experiment, our model parameters are only 10.62MB, while the MIMO-UNet network has a parameter amount of 25.97MB.

**Table 2:** Quantitative comparisons in terms of PSNR and SSIM (the symbol “↑” means that higher value is better) with state-of-the-art image dehazing, motion deblurring, and compression deblurring methods.

		Dehaze			MotionDeblur			Compression Deblur	
		PSNR↑	SSIM↑		PSNR↑	SSIM↑		PSNR↑	SSIM↑
Task-specific	AODNet	13.99	0.7592	MS-CNN	28.47	0.8107	ARCNN	27.62	0.7986
	GCANet	33.87	0.9632	SRN	28.35	0.7968	FBCNN	30,99	0.9001
	MSBDN	14.96	0.8864	DeblurGAN-v2	27.54	0.7752	HINet	**33.11**	**0.9104**
	FFANet	20.97	0.9325	DMPHN	29.59	0.8248	SADNet	32.81	0.9095
	YOLY	10.22	0.5284	MIMO-UNet	31.33	**0.9317**	MPRNet	32.47	0.8626
	**AIENet**	**35.69**	**0.9642**	**AIENet**	**31.87**	0.8648	**AIENet**	32.98	0.8764
All-in-one	**AIENet**	32.50	0.9501	**AIENet**	28.88	0.7909	**AIENet**	31.70	0.8587

The best and second-best results are highlighted in bold and underlined, respectively. The above half of the table shows comparisons of our task-specific models individually evaluated for each task. The last row of the table show evaluations of our all-in-one model AIENet on all three test sets.

**Figure 5:**
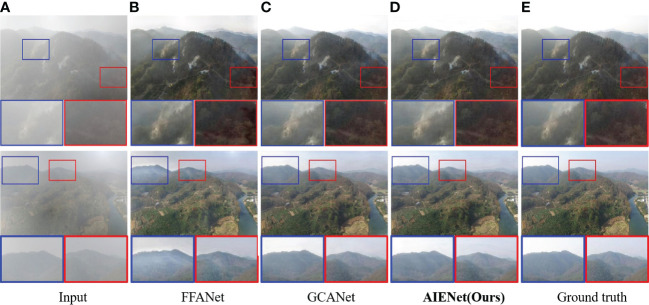
Qualitative enhancement comparisons of our model on synthetic hazy samples with FFANet (Qin et al., 2020) and GCANet (Ren et al., 2018). **(A)** is the input hazy images, **(B, C)** are the enhancement results of the state-of-the-art algorithms, **(D)** is the enhancement results of the proposed AIENet, and **(E)** is the ground truth images. Blue and red boxes correspond to the zoomed-in patch for better comparison.

**Qualitative Results for Image Dehazing.** To illustrate that our model can better remove the visual effects of haze and restore more image details than other dehazing methods, **Figure 5** depicts some visualizations of image dehazing reconstructions for aerial images of forests, comparing our method with FFANet (Qin et al., 2020) and GCANet (Ren et al., 2018). As illustrated, the FFANet does not completely remove the influence of haze, and its restored image has some artifacts. While GCANet seems to have the comparable visual quality to our model in image dehazing, our AIENet achieves visually pleasing results in detail enhancement (enlarged in red and blue bounding boxes).

**Figure 6:**
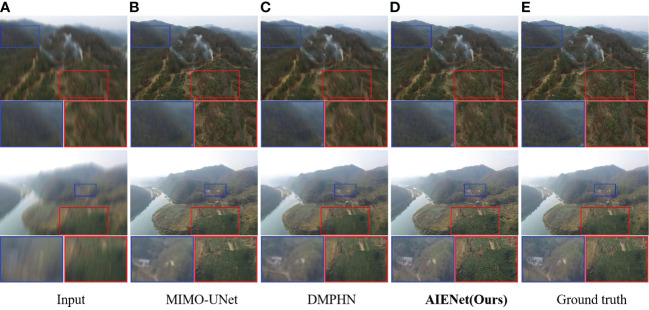
Qualitative enhancement comparisons of our model on synthetic motion blur samples with MIMO-UNet (Cho et al., 2021) and DMPHN (Zhang et al., 2019). **(A)** is the input motion blur images, **(B, C)** are the enhancement results of the state-of-the-art algorithms, **(D)** is the enhancement results of the proposed AIENet, and **(E)** is the ground truth images. Blue and red boxes correspond to the zoomed-in patch for better comparison.

**Figure 7:**
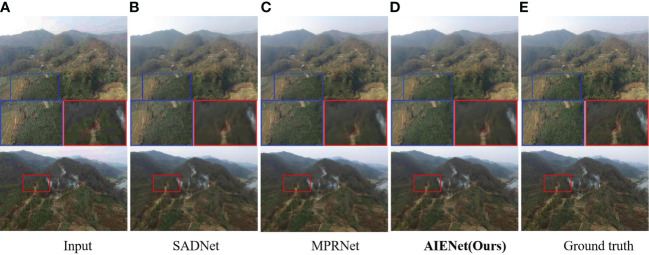
Qualitative enhancement comparisons of our model on synthetic compression blur samples with SADNet (Chang et al., 2020) and MPRNet (Zamir et al., 2021). **(A)** is the input compression blur images, **(B, C)** are the enhancement results of the state-of-the-art algorithms, **(D)** is the enhancement results of the proposed AIENet, and **(E)** is the ground truth images. Blue and red boxes correspond to the zoomed-in patch for better comparison.

**Qualitative Results for Image Deblurring:** To demonstrate the images restored by our model are sharper and produce fewer artifacts, **Figure 6** visualizes motion deblurring examples, demonstrating the superiority of our model AIENet over MIMO-UNet (Cho et al., 2021) and DMPHN (Zhang et al., 2019).In particular, the state-of-the-art methods still retain obvious streak artifacts when restoring images, while our model can preserve the structural and textural image details. (*e.g.*, second example in **Figure 6**, forest enlarged in the red bounding boxes). Although in quantitative experiments, the proposed model does not show competitive performance on image compression deblurring. But in visual analysis, as shown in **Figure 7**, the proposed model can produce excellent visual quality on par with state-of-the-art methods.

**Generality to Different Image Enhancement Tasks:** To demonstrate the superior generalization of the proposed method, we compare it with different task-specific image enhancement methods on three challenging tasks including dehaze, motion deblur and compression deblur. As shown in **Table 3**, although HINet performs well in removing compression artifacts, it has uncompetitive results on image dehazing and motion deblurring. Similarly, FFANet exhibits significant performance discrepancies across different tasks, with a PSNR of 32.17 dB for compression deblurring but only 20.97 dB for dehazing. Evidently, these methods excel only in specific tasks while performing poorly in others. Although generalization seems to be visible in MIMO-UNet, our AIENet exhibits a more competitive restoration performance than it in each task. The experimental results on datasets for hazy, motion blurring, and compression blurring show that our model excels at generalizing to diverse image domains.

**Table 3:** Quantitative comparison results (PSNR/SSIM) of some excellent methods for dehazy, motion deblurring and compression deblurring tasks.

Methods	Haze	Haze	Compression Blur
FFANet (Dehaze)	20.97/0.9325	29.60/0.8366	32.17/0.8785
FFANet (Dehaze)	30.12/**0.9842**	31.33/**0.9317**	32.76/**0.9408**
HINet (Compression Deblur)	21.94/0.9563	31.20/0.9035	**33.11**/0.9104
**Ours (All-in-one)**	32.50/0.9501	28.88/0.7909	31.70/0.8587
**Ours**	**35.69**/0.9642	**31.87**/0.864	32.98/0.8764

The best and second-best evaluation results are highlighted in bold and underlined, respectively.

#### 4.3.2 All-in-one image enhancement results

The last row of the **Table 2** presents quantitative evaluations for all-in-one image restoration. Generally, our method yields exceptional image quality and is faithful to the ground truth on all three test sets. Notably, for the image dehazing task, our trained all-in-one image enhancement network is second only to GCANet, the state-of-the-art model trained on the specific task, with PSNR/SSIM metrics reaching 32.50 dB/0.9501. Generally, the difference in image quality is less noticeable when the PSNR value reaches above 28 dB. Therefore, our model shows its outstanding performance and application value in environments that are sensitive to computational cost and running time.

### 4.4 Ablation study

In this section, we present ablation experiments to analyze the contribution of each component of our model. Specifically, we focus on two major components: with/without the skip connection and with the different number of enhancement blocks. Task-specific evaluation is performed on the synthetic haze dataset with the proposed models trained on the image size of 640Ã—640, and the results are shown in **Table 4**. To further validate the importance of each specific component in the all-in-one task, we also conduct analysis on the union of three datasets. Generally, we evaluate four different network configurations and follow the same training setup as the above experiments.

**Table 4:** Ablation study on individual components of the proposed AIENet.

Method		Task-specific				All-in-one			
		PSNR↑		SSIM↑		PSNR↑		SSIM↑	
*w/o* global-local skip connection		32.36	(10.3%)	0.9290	(3.8%)	30.11	(7.9%)	0.8664	(9.7%)
*w/o* local skip connection		33.96	(5.1%)	0.9553	(0.9%)	29.61	(4.8%)	0.8681	(2.2%)
MRF	*n* = 1	30.16	(18.3%)	0.9147	(5.4%)	30.85	(5.3%)	0.8839	(7.5%)
	*n* = 2	34.25	(4.2%)	0.9541	(1.1%)	31.02	(4.8%)	0.8840	(7.5%)
	*n* = 4	36.18	(-1.4%)	0.9651	(-0.1%)	31.69	(-2.1%)	0.8890	(-0.2%)
**Ours**		**35.69**	(0.0%)	**0.9642**	(0.0%)	**31.05**	(0.0%)	**0.8875**	(0.0%)

The PSNR and SSIM of the proposed method are highlighted in bold. The symbol “↑” means that higher value is better.

**Figure 8:**
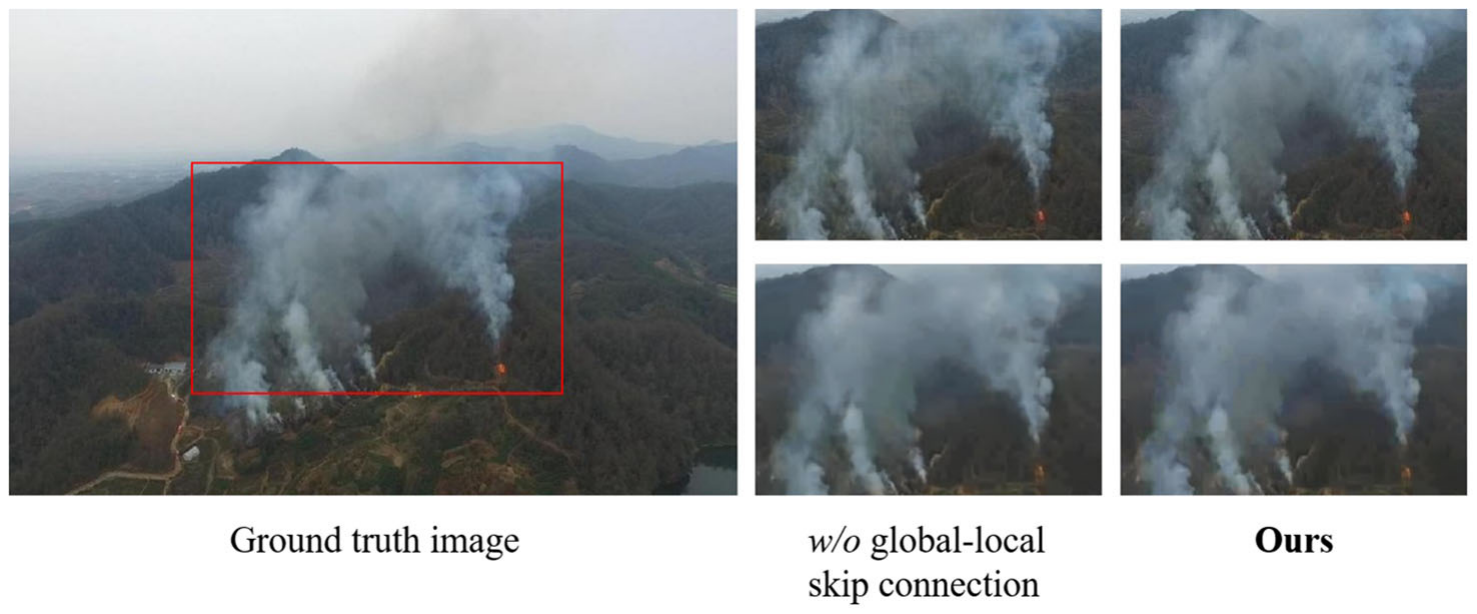
Examples on image dehazing(first row) and all-in-one image enhancement(second row) to show the superiority of global-local skip connection. Obviously, our model with glob-local skip connection improves the gridding artifacts and produces much better details.

**The influence of global-local skip connection:** As mentioned in Section 3.2, skip connection can provide more high-resolution details of the original image for deconvolution or upsampling processes. Therefore, we demonstrate the influence of the design by removing them from our final model. **Table 4** shows a substantial drop in PSNR of the image dehazing results from 35.69 dB to 32.36 dB when the global-local skip connection is removed. Correspondingly, the absence of the skip connection leads to poor performance as compared to employing it for all-in-one image enhancement. A similar trend is observed for the method without a local skip connection, where gains of the original model over it are 1.73 dB/0.0089 on PSNR/SSIM. We also provide two representative dehazing examples in **Figure 8** for visual comparison. It can be seen that the images restored by removing skip connections contain either overly smooth contents or artifacts with grid textures. In contrast, the complete model is able to remove real noise, while preserving the structural and textural image details.

**The effectiveness of the proposed MRF enhancement block:** Since our model could employ different enhancement block number, we test different options. The results on image dehazing and all-in-one image enhancement tasks corresponding to different *n* are given in **Table 4**. This ablation study reveals that MRF block effectively increases the PSNR by 13.56% from *n*=1 to *n*=2, owning to the diverse receptive fields and the multi-scale perception mechanism. It is worth noticing that the model yields better performance in PSNR and SSIM respectively as the number of the MRF enhancement block increases, but the gains show a clear downward trend. As such, the results also indicate that the model performance is not from the deeper layers but from a more efficient architecture, since more modules do not improve the performance much and our model has a smaller size.

### 4.5 Smoke detection results

**Figure 9:**
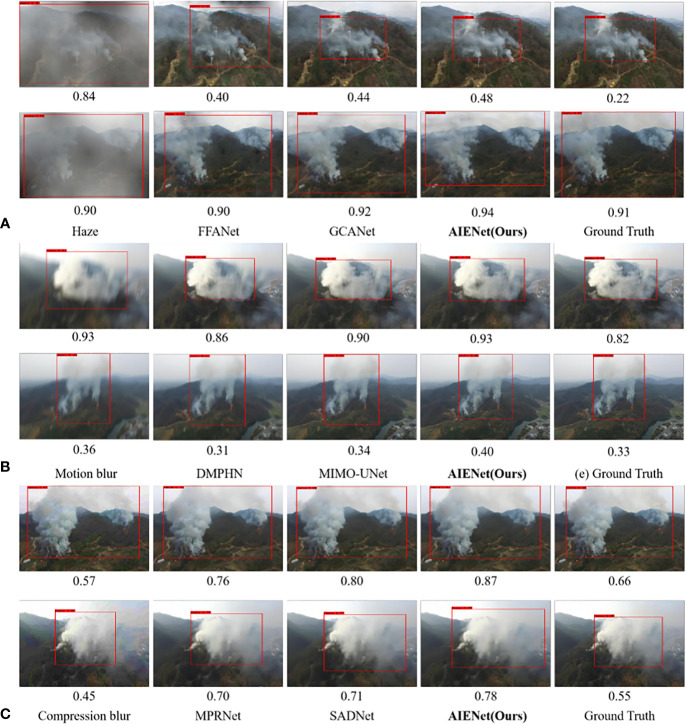
Qualitative comparison of our model with other works in improving fire detection performance. **(A)** is the visual comparison of image dehazing results. **(B)** is the visual comparison of image motion deblurring results. **(C)** is the visual comparison of image compression deblurring results.

As discussed in the introduction, aerial image enhancement could be helpful in improving the performance of fire detection approaches in forest fire prevention based on drone imagery monitoring. Therefore, we train a smoke detection algorithm (Wang et al., 2022) on the raw dataset. To verify the effectiveness of our method in boosting image detection, we use the results of image dehazing, image motion deblurring, and image compression deblurring as input exemplars for the detection algorithm, respectively. As a comparison, we also train models that perform better in each task, then test their enhanced results in the detection algorithm. As shown in **Figure 9**, the confidence below these images demonstrates the quantitative comparisons between the proposed model and the state-of-the-art methods. The results show that whether it is restoring blurred images or removing weather disturbances, our image enhancement method can effectively improve the confidence of the detection algorithm.

## 5 Conclusion

This paper presents an image enhancement method based on multiple receptive fields to improve the visual effect of aerial images in forest scenes. We focused on building an all-in-one framework that eliminates any degradation in aerial imagery. Based on this, we also devise a novel multi-receptive field enhancement block, which can adapt to the distribution differences of object regions in aerial images. It also benefits the network by recovering high-resolution details of images more efficiently. Extensive experiments have validated the merits of our method over other state-of-the-art enhancement methods on benchmark datasets. Specifically, our AIENet has achieved considerable gains in both dehazing and motion deblurring tasks, *i.e.*, 5.3% improvement in PSNR on the haze dataset, and a 1.7% increase on the motion blur dataset. The results of all-in-one image enhancement also show that our model has the ability to obtain performance close to SOTAs, which avoids the lack of resources associated with storing models separately to handle individual enhancement tasks. And we have also experimentally demonstrated that AIENet generalizes well to other image domains. Moreover, we further conduct ablation experiments to demonstrate the influence of the proposed MRF enhancement block. We show that using three enhancement blocks leads to optimal performance (35.69 dB on a specific task, and 31.05 dB on an all-in-one task) as compared to employing other quantities of enhancement blocks. Notably, the proposed method introduces lightweight image enhancement capability since the architecture can be based on a simpler backbone network for image restoration with less running time, which is of great interest for devices with limited resources.

## Data availability statement

The datasets presented in this study can be found in online repositories. The names of the repository/repositories and accession number(s) can be found in the article/supplementary material.

## Author contributions

ZC designed the experiments and wrote the first draft of the manuscript. CW made substantial contributions to the design of the study and the revision of the manuscript. FZ received financial support for this project and performed the analysis of the manuscript. LZ performed the experimental data preparation. AG and EG contributed to the revision of the manuscript, and read and approved the submitted version. All authors contributed to the article and approved the submitted version.
